# Karyotypes of two rare rodents, *Hapalomys delacouri* and *Typhlomys cinereus* (Mammalia, Rodentia), from Vietnam

**DOI:** 10.3897/zookeys.164.1785

**Published:** 2012-01-11

**Authors:** Alexei V. Abramov, Vladimir M. Aniskin, Viatcheslav V. Rozhnov

**Affiliations:** 1 Zoological Institute, Russian Academy of Sciences, Universitetskaya nab. 1, Saint-Petersburg 199034, Russia; 2 A.N. Severtsov Institute of Ecology and Evolution, Russian Academy of Sciences, Leninskii pr., 33, Moscow 119071, Russia; 3 Joint Vietnam-Russian Tropical Research and Technological Centre, Nguyen Van Huyen, Nghia Do, Cau Giay, Hanoi, Vietnam

**Keywords:** karyotypes, *Hapalomys delacouri*, *Hapalomys pasquieri*, *Typhlomys cinereus*, Vietnam

## Abstract

Karyotypes of *Hapalomys delacouri* (Rodentia, Muridae) and *Typhlomys cinereus* (Rodentia, Platacanthomyidae) from Vietnam are described for the first time. The diploid karyotype of *Hapalomys delacouri* is 38 (NFa=48), consisting of six pairs of bi-armed and 12 pairs of acrocentric autosomes decreasing in size; plus a large metacentric X chromosome and Y chromosome, also metacentric, that is equal in size to the largest pair of acrocentric autosomes. The newly described karyotype differs significantly from that reported for *Hapalomys delacouri* from northern Thailand. The latter record very likely represents a different species of *Hapalomys*, possibly the taxon *Hapalomys pasquieri* described from north-central Laos.The diploid karyotype of *Typhlomys cinereus* is 38 (NF=48), consisting of five pairs of meta- to submetacentric and 14 pairs of acrocentric chromosomes varying in size from large to small; sex chromosomes were not defined.

## Introduction

According to the recent checklist by Can et al. (2008), the mammal fauna of Vietnam consists of 295 species. During recent years, a half of dozen of new species have been found in Vietnam, including shrews, bats and a rodent (Jenkins et al. 2009, 2010, Borisenko et al. 2009, Tran et al. 2009, Bannikova et al. 2011). Rodents represent one of the most diverse but yet taxonomically neglected group of Vietnamese mammals.

It is well known that karyological data can be useful for tackling problems of rodent taxonomy and evolution (Volobouev et al. 2002, 2007, Aniskin et al. 2006, Kovalskaya et al. 2011). Therefore, cytotaxonomy represents an important step toward the inventory of the rodent species of Vietnam (Duncan et al. 1970, Cao and Tran 1985, Baskevich and Kuznetsov 1998). In the present paper, the karyotypes of two rare and poorly-known Vietnamese rodents – *Hapalomys delacouri* and *Typhlomys cinereus* – are described for the first time.

## Material and methods

**Figure 1.**
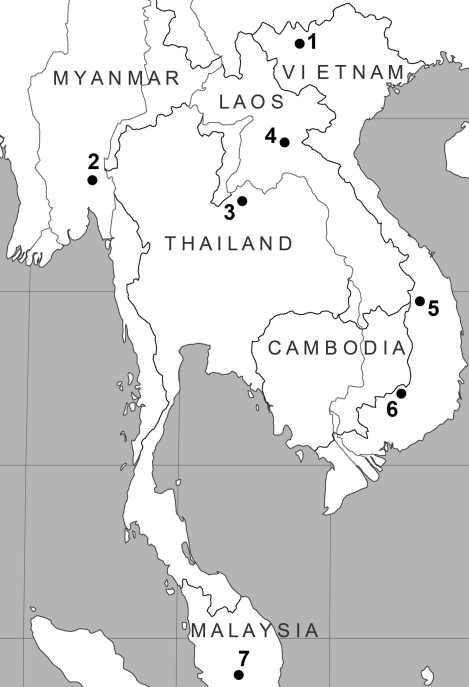
Map of localities. **1** sampling locality of *Typhlomys cinereus* **2** type locality of *Hapalomys longicaudatus* **3** locality from Badenhorst et al. 2009 **4** type locality of *Hapalomys pasquieri* **5** type locality of *Hapalomys delacouri* **6** sampling locality of *Hapalomys delacouri* in Bu Gia Map **7** approximate locality for *Hapalomys longicaudatus* record from Yong et al. 1982.

A number of rare and poorly-known mammal species were collected during a biodiversity surveys carried out by the Joint Vietnam-Russian Tropical Research and Technological Centre in 2010. Voucher specimens are deposited in the Zoological Institute of the Russian Academy of Sciences (ZIN), Saint-Petersburg, Russia. Five specimens of the marmoset rat *Hapalomys delacouri* Thomas, 1927 were collected in southern Vietnam, NE of Bu Gia Map Village, Binh Phuoc Province (12°12'N, 107°12'E; ZIN 98922, 99486-99488, 100410). A specimen of the soft-furred tree mouse *Typhlomys cinereus* Milne-Edwards, 1877 was collected in northern Vietnam, near Tram Ton Station of Hoang Lien National Park, W of Sa Pa Village, Lao Cai Province (22°21'N, 103°46'E; ZIN 100411). The collecting localities are shown in Fig.1.

The rodents were caught alive using locally made cage traps. The specimens were immediately brought to the laboratory where they were karyotyped. Chromosome analysis was carried out on preparations obtained from bone marrow following the standard colchicines method (Ford and Hamerton 1956). Slides were stained with 4% Giemsa in phosphate buffer with pH=6.8. At least 20 quality metaphases were analyzed for each specimen.

## Results and discussion

### *Hapalomys delacouri* Thomas, 1927 – lesser marmoset rat

**Figure 2.**
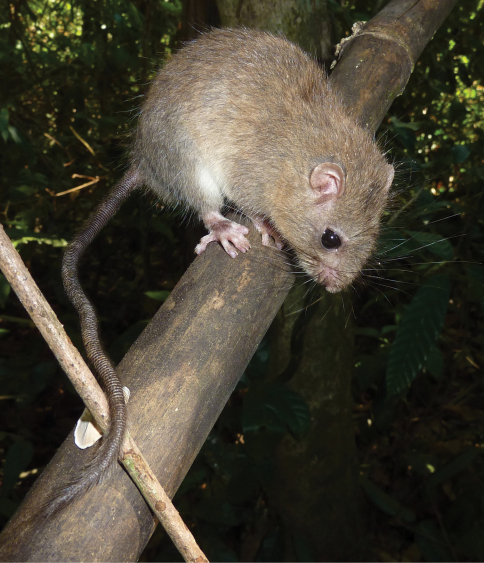
*Hapalomys delacouri*. Adult male from Bu Gia Map, Binh Phuoc Province, southern Vietnam. Photographed by Alexei V. Abramov.

The marmoset rats have very distinct external and cranial characteristics which preclude an incorrect generic identification (Thomas 1927, Musser 1972, Corbet and Hill 1992) – see Fig. 2.

**Figure 3.**
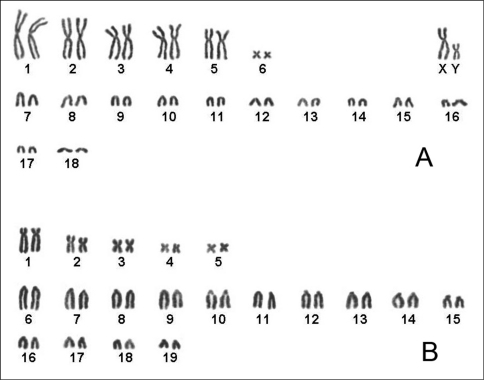
**A** Karyotype of male *Hapalomys delacouri* (ZIN 100410), 2n=38, NFa=48 **B** Karyotype of female *Typhlomys cinereus* (ZIN 100411), 2n=38, NF=48.

The diploid chromosome number is 2n=38, NFa=48 (Fig. 3A). This karyotype consists of six pairs of bi-armed and 12 pairs of acrocentric autosomes decreasing in size, with a large metacentric X chromosome and with Y chromosome, also metacentric, which is equal in size to the largest pair of acrocentric autosomes.

The observed karyotype differs significantly from that described by Badenhorst et al. (2009) for *Hapalomys delacouri* from Loei, northern Thailand (see Fig. 1). The latter authors reported the karyotype as having 2n=48 and NFa=92. All the autosomes were bi-armed (metacentric or submetacentric). The metacentric X and the acrocentric Y were easily recognizable because they were, respectively, the largest and the smallest elements in the karyotype. Earlier, Yong et al. (1982) described the karyotype of *Hapalomys longicaudatus* Blyth, 1859 based on a specimen from Malaysia. The diploid number of this specimen was 2n=50, consisting of 23 pairs of uniarmed and 1 pair of small bi-armed autosomes, metacentric X and subacrocentric Y sex chromosomes. The X chromosome was the largest element in the complement and constituted about 7.8% of the female haploid complement. The Y-chromosome was also distinct, being the only morphological type among the larger sized chromosomes, and constituted about 5.2% of the female haploid complement.

According to recent taxonomic studies (Musser and Carleton 1993, 2005, Nowak 1999), the genus *Hapalomys* consists of two species – *delacouri* and *longicaudatus* – distributed in eastern and western parts of Southeast Asia, respectively. The two species differ in coloration and size (Thomas 1927, Musser 1972, Corbet and Hill 1992). The specimens from Bu Gia Map are similar in coloration, size and body proportions (Table 1) to the lesser marmoset rat *Hapalomys delacouri*, which was described by Thomas (1927) from Kon Tum Province in southern Vietnam (Fig. 1). Another form of the marmoset rats was described by Thomas (1927) as *Hapalomys pasquieri* from Xieng Khouang in northern Laos (Fig. 1). Musser (1972) considered this form as a subspecies of *Hapalomys delacouri* based on similarities in coloration. Strong karyological differences between our specimen taken from southern Vietnam and the specimen from northern Thailand recorded by Badenhorst et al. (2009) point to a species level divergence. On distributional grounds (Fig. 1) the species in northern Thailand is most likely *Hapalomys pasquieri* but this needs confirmation by morphological comparison of the Thai and Laotian specimens. Further taxonomic studies of the genus *Hapalomys* are needed and cytotaxonomy can be a valuable tool for diagnosing the species involved.

**Table 1.** External and cranial measurements (range and means, in mm) of *Hapalomys* spp. The cranial measurements are explained in Musser (1970).

Measurements	*Hapalomys longicaudatus* (from Musser 1972), n=3-4	*Hapalomys delacouri* (from Musser 1972), n=4-5	*Hapalomys delacouri* Bu Gia Map, n=4	*Hapalomys pasquieri* (from Musser 1972), n=1
Length of head and body	162-165 (163.5)	123-136 (131.0)	130-146 (136.7)	121.0
Length of tail	193-202 (198.3)	140-160 (149.2)	155-165 (160.0)	171.0
Greatest length of skull	39.7-41.5 (40.47)	33.6-34.2 (34.00)	34.6-35.7 (35.10)	32.0
Length of nasals	11.5-12.6 (12.25)	11.7-12.0 (11.87)	11.7-11.9 (11.72)	10.5
Length of rostrum	9.7-10.2 (9.93)	9.3-9.7 (9.47)	9.4-10.0 (9.66)	8.3
Height of brain case	11.2-12.0 (11.63)	9.1-9.5 (9.33)	9.1-9.7 (9.49)	9.0
Palatal length	18.1-22.3 (20.78)	16.9-18.0 (17.48)	17.4-18.2 (17.83)	15.8
Maxillary tooth-row*	7.9-8.0	ca. 6.3	6.3-6.6 (6.4)	ca. 5.9

* Data from Corbet and Hill (1992) except for our specimens.

### *Typhlomys cinereus* Milne-Edwards, 1877 – soft-furred tree mouse

The diploid chromosome number is 2n=38, NF=48 (Fig. 3B), consisting of five pairs of meta- to submetacentric and 14 pairs of acrocentric chromosomes varying in size from large to small. Sex chromosomes of *Typhlomys cinereus* have not defined, as the female only was karyotyped in this study. It is the first karyotype described for a representative of the genus *Typhlomys*.

**Figure 4.**
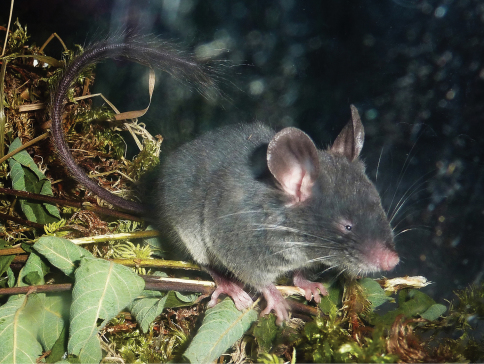
*Typhlomys cinereus*. Adult female from Sa Pa, Lao Cai Province, northern Vietnam. Photographed by Alexei V. Abramov.

The soft-furred tree mouse, or pygmy dormouse, *Typhlomys* *cinereus* (Fig. 4) belongs to the enigmatic family Platacanthomyidae, the earliest phylogenetic offshoot within Muroidea (Jansa et al. 2009). It is best knownfrom mountain forests of southern China, with an outlying population at high elevations in the northern part of Hoang Lien Mts in northern Vietnam (Nowak 1999, Musser and Carleton 2005, Can et al. 2008). The Vietnamese population was described as a separate species, *Typhlomys chapensis* (Osgood 1932) but it is now considered a subspecies of *Typhlomys cinereus* (Musser and Carleton 2005). Further morphological and genetic studies are needed to clarify the taxonomic status of the Vietnamese soft-furred tree mouse.
